# Dr. W. B. Preston

**Published:** 1905-03

**Authors:** 


DR. W. B. PRESTON, of Dansville, N. Y., died at his home, February 4, 1905, aged 60 years. He graduated at the Eclectic Medical Institute, Cincinnati, in 1867, and had been ill of Bright's disease for more than a year.

